# Stability of Non-Linear Dirichlet Problems with *ϕ*-Laplacian

**DOI:** 10.3390/e23060647

**Published:** 2021-05-22

**Authors:** Michał Bełdziński, Marek Galewski, Igor Kossowski

**Affiliations:** Institute of Mathematics, Lodz University of Technology, Wólczańska 215, 90-924 Lodz, Poland; michal.beldzinski@dokt.p.lodz.pl (M.B.); igor.kossowski@p.lodz.pl (I.K.)

**Keywords:** Browder–Minty Theorem, Dirichlet BVP, Hadamard Programme, *ϕ*-Laplacian, stability of solution

## Abstract

We study the stability and the solvability of a family of problems $-{\left(\phi \left(x^{\prime }\right)\right)}^{\prime }=g\left(t,x,x^{\prime },u\right)+f^{*}$ with Dirichlet boundary conditions, where ϕ, *u*, $f^{*}$ are allowed to vary as well. Applications for boundary value problems involving the *p*-Laplacian operator are highlighted.

## 1. Introduction

The Hadamard Programme about non-linear equations concerns the following:

(a) the solvability;
(b) the uniqueness;
(c) the dependence on parameters.

Note that (c) can be viewed (and is sometimes called) as a type of a stability, which is not to be confused with the Lyapunov stability as described in [1]. The issues named as (a) and (b) have been widely considered by the non-linear analysis methods (the variational method, the usage of critical point theory, the method of monotone operators, the degree theory, various fixed point results), see for example [2,3,4,5] to mention books covering the existence tools pertaining to the method of monotone operators applied here. Apart from the major reference books mentioned, there are a number of recent results dealing with the not necessarily variational existence of boundary value problems, and also with a type of approximation leading to the solvability of a given problem. Let us mention, without being exhaustive, for example, [6] where the celebrated Leray–Lions Theorem is utilized in order to generate a sequence which further approximates the solution to the Dirichlet problem with the $p\left(x\right)$-Laplacian. In [7], the Leray–Schauder degree is used to investigate the equations on integers governed by the $p\left(k\right)$-Laplacian which may further serve as an approximating sequence to some boundary value problem. Problems driven by the ϕ-Laplacian were investigated by the Harnack inequality, combined with fixed point approaches pertaining to the Bohnenblust–Karlin fixed point theorem in [8] and the Schauder, the Krasnosel’skii fixed point theorems in [9]. Boundary value problems for equations and systems with the *p*-Laplacian, as well as bounded or singular homeomorphisms are considered by the Krasnosel’skii type compression–expansion arguments and by a weak Harnack type inequality in [10].

On the other hand, the third issue has not been given that much attention, we can mention [11] describing the variational approach towards the dependence on parameters and also [12] where monotonicity methods are used. Some abstract scheme best reflecting the type of stability applied here allowing for various parameters is to be found in [13], where stability or well-posedness results are proved for families of semi-linear operator equations. There was also some research relating the dependence on eigenvalues of the Dirichlet problem with the *p*-Laplacian as *p* varies. All these sources mentioned employ the uniform bound on the sequence of solutions together with their weak characterization and suitable embedding results. There is also research in a different direction, which not only reflects the dependence on parameters. Namely, in [14] it is considered the convergence of eigenvalues of the *p*-Laplacian as p→1 by using approximation of BV(Ω) functions by $C_{0}^{\infty }\left(\Omega \right)$ functions in the sense of strict convergence on $R^{n}$. Paper [15] concerns the case of the variational eigenvalues of the $p\left(x\right)$-Laplacian under the uniform convergence of the exponents investigated by variational methods. We mention also the recent [16] which treats problems with the right hand side independent of the sought function which investigates the dependence of gradients of solutions as p→∞. In this paper, we are concerned with the dependence on non-linear functional parameters for problems governed by the *p*-Laplacian also with *p* being treated as a numerical parameter. Contrary to [11,12,13] we do not concentrate only on problems governed by the (negative) Laplacian but include the boundary problems driven by *p*-laplacian for p>2 into our consideration. Moreover, the approach towards the stability is based not on the investigation of the sequence of solutions corresponding to the sequence of parameters but on the analysis of the solution operators which makes our main stability result, namely Theorem 4, independent of the existence method (among mentioned above) which is employed in order to prove the solvability of the relevant (non-linear) equation. We allow for p→2 due to tools which we apply for the solvability. An easy example best illustrating what sort of problems we may encounter now follows.

**Example** **1.** *Let us consider for n∈N the following family of Dirichlet problems with $p_{n}>2$ and $\lambda_{n}>\pi^{2}$:*

(1) $$\left\{\begin{array}{c} -{\left(|x^{\prime }{|}^{p_{n}-2}x^{\prime }\right)}^{\prime }=\lambda_{n}x+\sin\left(\pi t\right),\\ x(0)=x(1)=0. \end{array}\right.$$

*Note that for every n∈N there exists a solution $x_{n}$ to *(1)*. If we let $p_{n}↘p_{\infty }=2$ and $\lambda_{n}↘\lambda_{\infty }=\pi^{2}$ then it is direct to observe that *(1)* is unsolvable with $p_{\infty }$, $\lambda_{\infty }$, see [17] for details. This observation we supply with the following additional conclusions: $\left(x_{n}\right)$ is not bounded in $W_{0}^{1,2}\left(0,1\right)$ which means that it is not weakly convergent up to a subsequence, hence it is not (weakly) compact in $W_{0}^{1,2}\left(0,1\right)$.*

In what follows we will provide some general conditions which exclude the phenomena appearing above, as well as conditions on $p_{\infty },\lambda_{\infty }$ under which one obtains the convergence in Example 1. The paper is organized as follows. We start with some preliminaries about functional space setting, illustrating the relations between spaces involved by some figure and providing some version of the well known Krasnosel’skii Theorem on the continuity of the Niemytskij operator, as well as some general stability results. Boundary value problems with the ϕ-Laplacian are next considered with the right hand side independent of the sought function and for which the existence and stability result. The existence is reached by a direct formula exploiting the properties of the increasing homeomorphism and the stability is obtained by investigating the continuity of the solution operator. Next, with the aid of the Browder–Minty Theorem such results are shifted to problems containing non-linear perturbations. Examples and comments are included into the text, corresponding also to the Dirac delta thus showing the possible general applicability of our results.

## 2. Preliminaries and Auxiliary Results

Following [17] we denote by $W^{1,p}\left(0,1\right)$, p∈[1,∞), the space of all absolutely continuous functions with $L^{p}$-integrable derivative. For another approach towards the Sobolev spaces on [0,1], see [18]. We refer is the sequel to both sources for the background. If not said otherwise, we consider any p≥1. We endow $W_{0}^{1,p}\left(0,1\right)$ with a standard norm

$${∥x∥}_{W^{1,p}}={\left(\int_{0}^{1}{\left|x\left(t\right)\right|}^{p}dt+\int_{0}^{1}{\left|x^{\prime }\left(t\right)\right|}^{p}dt\right)}^{1/p}.$$

Recall that inclusion $W^{1,p}\left(0,1\right)\subset C\left[0,1\right]$ is continuous for every *p* and compact if p>1. We denote

$$W_{0}^{1,p}\left(0,1\right):=\left\{x\in W^{1,p}\left(0,1\right):x\left(0\right)=x\left(1\right)=0\right\}$$

and consider it with a norm

$${∥x∥}_{W_{0}^{1,p}}:={\left(\int_{0}^{1}{\left|x^{\prime }\left(t\right)\right|}^{p}dt\right)}^{1/p}$$

equivalent with ${∥\cdot ∥}_{W^{1,p}}$ on $W_{0}^{1,p}\left(0,1\right)$. A continuous dual of $W_{0}^{1,p}\left(0,1\right)$ will be denoted by $W^{-1,q}\left(0,1\right)$, here and in the sequel *q* is the Hölder conjugate to *p*, that is $\frac{1}{p}+\frac{1}{q}=1$. We put $\frac{1}{\infty }=0$ and denote a continuous dual of $W_{0}^{1,1}\left(0,1\right)$ by $W^{-1,\infty }\left(0,1\right)$. The *Poincaré* and the *Sobolev inequalities* are as follows: for all $x\in W_{0}^{1,p}$ we have

$${∥x∥}_{L^{p}}^{p}\leq \frac{1}{\lambda_{p}}{∥x∥}_{W_{0}^{1,p}}^{p}{\;\mathrm{and}\;∥x∥}_{C}\leq {∥x∥}_{W_{0}^{1,p}},$$

where ${∥x∥}_{C}=\max_{0\leq t\leq 1}\left|x\left(t\right)\right|$ and $\lambda_{p}=\inf_{x\in W_{0}^{1,p}\setminus \left\{0\right\}}{∥x∥}_{W_{0}^{1,p}}/{∥x∥}_{L^{p}}$ is the optimal constant in the Poincaré inequality. Ref. [19] [Chapter 1, Section 4] contains detailed calculations of this constant. The mapping $p⟼\lambda_{p}$ is increasing and continuous on (1,∞) and $\lambda_{2}=\pi^{2}$. Notice that as an immediate consequence of the Sobolev inequality we obtain

$$∥f^{*}{∥}_{W^{-1,q}}\leq {∥f^{*}∥}_{C^{*}}\;\;\mathrm{for}\;\mathrm{all}\;f^{*}\in {\left(C\left[0,1\right]\right)}^{*}\;\mathrm{and}\;\mathrm{every}\;q\in \left(1,\infty \right]$$

where ${∥\cdot ∥}_{C^{*}}$ stands for a norm in ${\left(C\left[0,1\right]\right)}^{*}$. Denote by *j* an continuous embedding $L^{1}\left(0,1\right)↪{\left(C\left[0,1\right]\right)}^{*}$ given by

(2) $$\langle jf,x\rangle =\int_{0}^{1}f\left(t\right)x\left(t\right)dt\;\mathrm{for}\;\mathrm{all}\;x\in C\left[0,1\right]$$

Functional $f^{*}\in {\left(C\left[0,1\right]\right)}^{*}$ is called *regular* if $f^{*}=jf$ for some $f\in L^{1}\left(0,1\right)$. We denote

$$L_{0}^{p}\left(0,1\right)=\left\{x\in L^{p}\left(0,1\right):\int_{0}^{1}x\left(t\right)dt=0\right\}.$$

**Figure 1:**
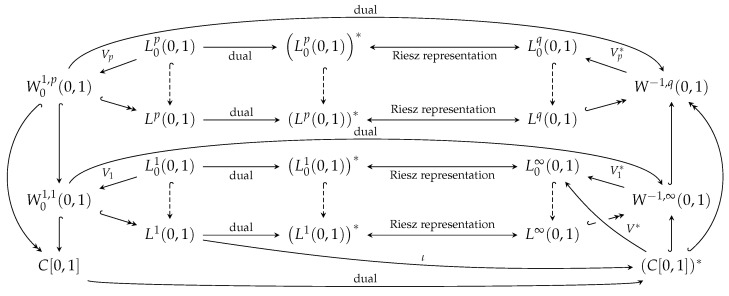
Dashed ↪ denotes an inclusion, ↪—continuous and dense inclusion, ↪→—a compact and dense inclusion.

Identifying ${\left(L^{p}\left(0,1\right)\right)}^{*}≃L^{q}\left(0,1\right)$ via the Riesz’ Representation Theorem we get ${\left(L_{0}^{p}\left(0,1\right)\right)}^{*}≃L_{0}^{q}\left(0,1\right)$. Moreover, a mapping $V_{p}:L_{0}^{p}\left(0,1\right)⟶W_{0}^{1,p}\left(0,1\right)$ defined by

$$\left(V_{p}x\right)\left(t\right):=\int_{t}^{1}x\left(s\right)ds\;\mathrm{for}\;\mathrm{all}\;t\in \left[0,1\right].$$

is an isometry between $L_{0}^{p}\left(0,1\right)$ and $W_{0}^{1,p}\left(0,1\right)$. Hence $V_{p}^{*}$ is an isometry between $L_{0}^{q}\left(0,1\right)$ (identified with a dual of $L_{0}^{p}\left(0,1\right)$) and $W^{-1,q}\left(0,1\right)$. Notice that $V_{p_{1}}^{*}f^{*}=V_{p_{2}}^{*}f^{*}$ for all $f^{*}\in {\left(C\left[0,1\right]\right)}^{*}\subset W^{-1,q_{1}}\left(0,1\right)\cap W^{-1,q_{2}}\left(0,1\right)$ and that

(3) $$\left(V_{p}^{*}f^{*}\right)\left(t\right)=\int_{0}^{t}f\left(s\right)ds\;\mathrm{for}\;\mathrm{all}\;t\in \left[0,1\right].$$

for every regular *f* and any *p*. Moreover, continuous inclusion $W^{1,1}\left(0,1\right)\subset C\left[0,1\right]$ provides that $V_{p}^{*}{\left(C\left[0,1\right]\right)}^{*}\subset L^{\infty }\left(0,1\right)$ for every *p*. Therefore, we can define a continuous linear operator $V^{*}:{\left(C\left[0,1\right]\right)}^{*}⟶L^{\infty }\left(0,1\right)$ using a formula $V^{*}f^{*}=V_{1}^{*}f^{*}$, which coincides with the Formula (3) for every regular *f*. Let us observe that compact inclusion $W^{1,p}\left(0,1\right)\subset C\left[0,1\right]$, p>1, provides that $f_{n}^{*}{⇀}^{*}f_{\infty }^{*}\Rightarrow V^{*}f_{n}^{*}\to V^{*}f_{\infty }^{*}$ in $L^{p}\left(0,1\right)$. Here $f_{n}^{*}{⇀}^{*}f_{\infty }^{*}$ denotes a weak* convergence of sequence $\left(f_{n}^{*}\right)$ to element $f_{\infty }^{*}$. From now on we equip ${\left(C\left[0,1\right]\right)}^{*}$ with a weak* topology. These relations are summarized in Figure 1.

Now we turn to the Niemytskii operator. For a closed set U⊂R denote

$$L^{\infty }\left(0,1;U\right):=\left\{u\in L^{\infty }\left(0,1\right):u\left(t\right)\in U\;\mathrm{for}\;a.e.\;t\in \left[0,1\right]\right\}.$$

Let $g:\left[0,1\right]\times R^{2}\times U⟶R$ be a *Carthéodory function*, that is:

- g(·,x,y,u) is Lebesgue-measurable for all $\left(x,y,u\right)\in R^{2}\times U$;
- g(t,·,·,·) is continuous for a.e. t∈[0,1].

The following result is a direct consequence of the Lebesgue Dominated Convergence Theorem.

**Theorem** **1.** *Assume that $x_{n}\to x_{\infty }$, $y_{n}\to y_{\infty }$ and $u_{n}\to u_{\infty }$, all in $L^{1}\left(0,1\right)$. If there exists a function $\zeta \in L^{p}\left(0,1\right)$ such that*

(4) $$\left|g\left(t,x_{n}\left(t\right),y_{n}\left(t\right),u_{n}\left(t\right)\right)\right|\leq \zeta \left(t\right)\;\;\mathrm{for}\;\mathrm{all}\;n\in N_{\infty }\;\mathrm{and}\;a.e.\;t\in \left[0,1\right],$$

*then $g\left(\cdot ,x_{n}\left(\cdot \right),y_{n}\left(\cdot \right),u_{n}\left(\cdot \right)\right)\to g\left(\cdot ,x_{\infty }\left(\cdot \right),y_{\infty }\left(\cdot \right),u_{\infty }\left(\cdot \right)\right)$ in $L^{p}\left(0,1\right)$.*

**Proof.**  Without lost of generality we can assume that $x_{n}\to x_{\infty }$, $y_{n}\to y_{\infty }$ and $u_{n}\to u_{\infty }$ pointwisely a.e. on [0,1]. Therefore, assumed continuity of g(t,·,·,·) provides

$${\left|g\left(t,x_{n}\left(t\right),y_{n}\left(t\right),u_{n}\left(t\right)\right)-g\left(t,x_{\infty }\left(t\right),y_{\infty }\left(t\right),u_{\infty }\left(t\right)\right)\right|}^{p}\to 0\;\mathrm{for}\;a.e.\;t\in \left[0,1\right].$$

Finally, the assumption (4) allows us to apply the Lebesgue Dominated Convergence Theorem. □

Let us consider a compact metric space Σ and a Banach space *X*. Let A:Σ×X⟶X be a continuous mapping such that for every bounded set B⊂X, the set A(Σ×B) is relatively compact in *X*. We denote

$$S_{\sigma }:=\left\{x\in X:A(\sigma ,x)=x\right\}\;\mathrm{and}\;B_{r}:=\left\{x\in X:∥x∥\leq r\right\}.$$

**Lemma** **1.** *Assume that A satisfies conditions given above and let $\sigma_{n}\to \sigma_{\infty }$ in *Σ*. Then every sequence $(x_{n})\subset X$ such that $x_{n}\in S_{\sigma_{n}}$ for n∈N, either has a subsequence convergent to some $x_{\infty }\in S_{\sigma_{\infty }}$ or else it holds satisfy $∥x_{n}∥\to \infty$.*

**Proof.**  Define $S={⋃}_{\sigma \in \Sigma }S_{\sigma }$. The continuity of *A* tells us that *S* is closed. Let $S^{r}:=S\cap B_{r}$. Since $S^{r}\subset A\left(\Sigma \times S^{r}\right)$, it follows that sets $S^{r}$ are compact for all r>0. Hence, whenever $\sigma_{n}\to \sigma_{\infty }$ and $x_{n}\in S_{\sigma_{n}}$, every bounded subsequence of $\left(x_{n}\right)$ has a further subsequence such that $x_{n}\to x\in S$. The continuity of *A* allows us to conclude that $x\in S_{\sigma_{\infty }}$. □

The results obtained can be expressed in terms of the upper semi-continuity of multi-valued mappings, see [20]. However we do not need such a general approach here.

Now, let us consider a real, reflexive and separable Banach space *X*. Operator $T:X⟶X^{*}$ is called *coercive* if

$$\lim_{∥x∥\to \infty }\frac{\langle T(x),x\rangle }{∥x∥}=\infty .$$

*T* is *monotone* if

〈T(x)−T(y),x−y〉≥0forallx,y∈X

and *T* is *strongly continuous* if $x_{n}⇀x_{\infty }$ in *X* implies that $T\left(x_{n}\right)\to T\left(x_{\infty }\right)$ in $X^{*}$. We say that *T* is *bounded* if T(B) is bounded in $X^{*}$ for every bounded B⊂X.

**Theorem** **2** ([21]). Assume that $T:X⟶X^{*}$ is continuous, coercive, and bounded. If additionally $T=T_{1}-T_{2}$, where $T_{1}$ is monotone and $T_{2}$ is strongly continuous, then $T\left(X\right)=X^{*}$.

## 3. Boundary Value Problems with the ϕ-Laplacian

We denote by Homeo(R) the space of all homeomorphism of the real line, equipped with a *topology of almost uniform convergence*, namely, we write $\phi_{n}\to \phi_{\infty }$ if and only if $\phi_{n}{{|}_{K}⇉\phi_{\infty }|}_{K}$ for every compact K⊂R. Direct calculations provide that $\phi_{n}^{-1}\to \phi_{\infty }^{-1}$ whenever $\phi_{n}\to \phi_{\infty }$. We will consider $N_{\infty }=N\cup \left\{\infty \right\}$ with a distance d(n,m)=|arctan(n)−arctan(m)|, where arctan(∞)=π/2. Thus it makes sense to write n=∞ with no confusion. For a fixed ϕ∈Homeo(R) and fixed $f^{*}\in {\left(C\left[0,1\right]\right)}^{*}$ we consider

(5) $$\left\{\begin{array}{c} -{\left(\phi \left(x^{\prime }\right)\right)}^{\prime }=f^{*},\\ x(0)=x(1)=0, \end{array}\right.$$

Solutions to (5) are understood in the following sense: an absolutely continuous function x:[0,1]⟶R is a solution to (5) if x(0)=x(1)=0, if $\phi (x^{\prime }\left(\cdot \right))$ is integrable and if

(6) $$\int_{0}^{1}\phi \left(x^{\prime }\left(t\right)\right)\chi^{\prime }\left(t\right)dt=\langle f^{*},\chi \rangle \;\mathrm{for}\;\mathrm{all}\;\chi \in C_{0}^{\infty }\left(0,1\right)$$

Notice that approach introduced covers also classical cases where the right hand side of (5) is continuous or integrable.

**Example** **2.** The assumption about $f^{*}\in {\left(C\left[0,1\right]\right)}^{*}$ allows us to consider problems of the form

$$\left\{\begin{array}{c} -{\left(|x^{\prime }{|}^{p-2}x^{\prime }\right)}^{\prime }=f\left(t\right)+\delta_{t_{0}}+\cdots +\delta_{t_{m}},\\ x(0)=x(1)=0, \end{array}\right.$$

*where $f\in L^{1}\left(0,1\right)$, $t_{0},\cdots ,t_{m}\in \left[0,1\right]$ and $\delta_{t}$ is* the Diracs delta*, that is*

$$\langle \delta_{t},x\rangle =x\left(t\right)\;\mathrm{for}\;\mathrm{every}\;x\in C\left[0,1\right].$$

Now we follow with a stability result which best reflects how we understand the dependence on parameters.

**Theorem** **3.** Let $f_{n}^{*}{⇀}^{*}f_{\infty }^{*}$ in ${\left(C\left[0,1\right]\right)}^{*}$ and $\phi_{n}\to \phi_{\infty }$ in Homeo(R). Then problem (5)* (with $\phi =\phi_{n}$ and $f=f_{n}$) has a unique solution $x_{n}$ for each $n\in N_{\infty }$. Moreover, $x_{n}\to x_{\infty }$ in $W_{0}^{1,p}\left(0,1\right)$ for every p.*

For the proof of Theorem 3 we need an auxiliary lemma, which describes properties of the solution operator. Note that we can replace ${\left(C\left[0,1\right]\right)}^{*}$ with $L^{1}\left(0,1\right)$, equipped with a weak topology, using the continuous embedding $L^{1}\left(0,1\right)↪{\left(C\left[0,1\right]\right)}^{*}$ and retain all the assertions.

**Lemma** **2.** For each $f^{*}\in {\left(C\left[0,1\right]\right)}^{*}$ and for every ϕ∈Homeo(R), there exists a unique $c=c(\phi ,f^{*})$ satisfying

(7) $$F_{\left(\phi ,f^{*}\right)}\left(c\right)=\int_{0}^{1}\phi^{-1}\left(c-\left(V^{*}f^{*}\right)\left(s\right)\right)ds=0.$$

Moreover, for every bounded sets K⊂Homeo(R) and every norm-bounded set $B^{*}\subset {\left(C\left[0,1\right]\right)}^{*}$, a mapping $c:K\times B^{*}∋\left(\phi ,f\right)⟼c\left(\phi ,f\right)\in R$ is bounded and continuous.

**Proof.**  For all $f^{*}\in {\left(C\left[0,1\right]\right)}^{*}$ and ϕ∈Homeo(R) we define $F_{\left(\phi ,f^{*}\right)}:R⟶R$ by (7). Function $F_{\left(\phi ,f^{*}\right)}$ is continuous, strictly monotone and $\lim_{|c|\to \infty }\left|F_{\left(\phi ,f^{*}\right)}\left(c\right)\right|=\infty$. Hence $F_{\left(\phi ,f^{*}\right)}\left(c\right)=0$ for a unique $c=c(\phi ,f^{*})$. Notice that for every increasing ϕ we have

$$\begin{array}{cc} \phi^{-1}\left(c\left(\phi ,f^{*}\right)-{∥f^{*}∥}_{C^{*}}\right) & \leq \int_{0}^{1}\phi^{-1}\left(c\left(\phi ,f^{*}\right)-\left(V^{*}f^{*}\right)\left(s\right)\right)ds\\ \; & \leq \phi^{-1}\left(c\left(\phi ,f^{*}\right)+{∥f^{*}∥}_{C^{*}}\right), \end{array}$$

since $V^{*}f^{*}\in L^{\infty }\left(0,1\right)$ and since $∥V^{*}f^{*}{∥}_{L^{\infty }}=∥f^{*}{∥}_{W^{-1,\infty }}\leq {∥f^{*}∥}_{C^{*}}$. Hence, the boundedness of *c* on $K\times B^{*}$ follows from inequalities

$$\phi \left(0\right)-∥f^{*}{∥}_{C^{*}}\leq c\left(\phi ,f^{*}\right)\leq \phi \left(0\right)+{∥f^{*}∥}_{C^{*}},$$

which hold for every ϕ. Now, let $\phi_{n}\to \phi_{\infty }$ in *K* and $f_{n}^{*}{⇀}^{*}f_{\infty }^{*}$ in $B^{*}$. Then $c(\phi_{n},f_{n}^{*})\to c$ up to a subsequence (not renumbered). Moreover, $V^{*}f_{n}^{*}\to V^{*}f_{\infty }^{*}$ in $L^{q}\left(0,1\right)$ and the sequence $\left(V^{*}f_{n}^{*}\right)$ is a.e. uniformly bounded. Hence we can use Theorem 1 to get $\phi_{n}^{-1}\left(c\left(\phi ,f_{n}^{*}\right)-\left(V^{*}f_{n}^{*}\right)\left(\cdot \right)\right)\to \phi_{\infty }^{-1}\left(c-\left(V^{*}f_{\infty }^{*}\right)\left(\cdot \right)\right)$ in $L^{p}\left(0,1\right)$ for every *p*. Then

$$0=\lim_{n\to \infty }\int_{0}^{1}\phi_{n}^{-1}\left(c\left(\phi ,f_{n}^{*}\right)-\left(V^{*}f_{n}^{*}\right)\left(s\right)\right)ds=\int_{0}^{1}\phi_{\infty }^{-1}\left(c-\left(V^{*}f_{\infty }^{*}\right)\left(s\right)\right)ds$$

and hence $c=c(\phi_{\infty },f_{\infty }^{*})$. This proves the continuity of *c*. □

Since $V^{*}f^{*}\in L^{\infty }\left(0,1\right)$ for all $f^{*}\in {\left(C\left[0,1\right]\right)}^{*}$, using Lemma 2 we obtain a classical solution to (5):

(8) $$x\left(t\right)=\int_{0}^{t}\phi^{-1}\left(c\left(\phi ,f^{*}\right)-\left(V^{*}f^{*}\right)\left(s\right)\right)ds.$$

Therefore, in our setting, notions of weak and classical solution overlap. Now we are in the position to proceed with the proof of main result.

**Proof of Theorem** **3** Formula (8) defines a solution to (5) for every fixed ϕ∈Homeo(R). Uniqueness of such a solution follows from simple calculation. Now, let $\phi_{n}\to \phi_{\infty }$ and $f_{n}^{*}{⇀}^{*}f_{\infty }^{*}$ in ${\left(C\left[0,1\right]\right)}^{*}$. Then arguing like in proof of Lemma 2 we obtain $\phi_{n}^{-1}\left(c\left(\phi ,f_{n}^{*}\right)-\left(V^{*}f_{n}^{*}\right)\left(\cdot \right)\right)\to \phi_{\infty }^{-1}\left(c\left(\phi_{\infty },f_{\infty }^{*}\right)-\left(V^{*}f_{\infty }^{*}\right)\left(\cdot \right)\right)$ in $L^{p}\left(0,1\right)$ for arbitrary *p*. But this is equivalent with $x_{n}\to x_{\infty }$ in $W_{0}^{1,p}\left(0,1\right)$. □

**Remark** **1.** If functionals $f_{n}^{*}$ are regular, that is $f_{n}^{*}=jf_{n}$ for $n\in N_{\infty }$, then assuming $f_{n}\to f_{\infty }$ in $L^{1}\left(0,1\right)$, we get $x_{n}\to x_{\infty }$ in $C^{1}\left[0,1\right]$. One can deduce it easily from (8) bearing in mind that in such a case

$$\left(V^{*}f_{n}^{*}\right)\left(t\right)=\int_{0}^{t}f\left(s\right)ds.$$

## 4. Non-Linear Perturbations of the ϕ-Laplcian Problems

Take a closed set U⊂R. We consider the following assumption.

(9) $$\left.\begin{array}{c} \phi_{n}\to \phi_{\infty }\;\mathrm{in}\;\mathrm{Homeo}\left(R\right),\\ u_{n}\to u_{\infty }\;\mathrm{in}\;L^{\infty }\left(0,1;U\right),\\ f_{n}^{*}{⇀}^{*}f_{\infty }^{*}\;\mathrm{in}\;{\left(C\left[0,1\right]\right)}^{*}. \end{array}\right\}$$

(10) $$\left.\begin{array}{c} g:\left[0,1\right]\times R^{2}\times U⟶R\;\mathrm{is}\;a\;\mathrm{Carth}é\mathrm{odory}\;\mathrm{function}.\\ \mathrm{There}\;\mathrm{exists}\;b>0,\rho \geq 1,a\in L^{1}\left(0,1\right)\;\mathrm{and}\;h\in C\left(R\times U\right)\;\mathrm{such}\;\mathrm{that}\;\\ \;|g\left(t,x,y,u\right)|\leq a\left(t\right)h\left(x,u\right)+{b|y|}^{\rho }\\ \mathrm{for}\;a.e.\;t\in \left[0,1\right]\;\mathrm{and}\;\mathrm{all}\;\left(x,y,u\right)\in R^{2}\times U. \end{array}\right\}$$

**Theorem** **4.** *Let assumption *(9)* and *(10)* holds. Assume that for every n∈N the problem*

(11) $$\left\{\begin{array}{c} {\left(\phi_{n}\left(x^{\prime }\left(t\right)\right)\right)}^{\prime }=g\left(t,x\left(t\right),x^{\prime }\left(t\right),u_{n}\left(t\right)\right)+f_{n}^{*},\\ x(0)=x(1)=0, \end{array}\right.$$

*has a solution $x_{n}\in W_{0}^{1,\rho }\left(0,1\right)$ understood in a weak sense (as in formula *(6)*) and not necessarily unique. Then*

*1*. *$∥x_{n}{∥}_{W_{0}^{1,\rho }}\to \infty$ whenever problem *(11)* (with n=∞) is unsolvable;*
*2*. *if sequence $\left(x_{n}\right)$ is bounded in $W_{0}^{1,p}\left(0,1\right)$ for some p≥ρ, then problem *(11)* (with n=∞) has a solution $x_{\infty }$ such that $x_{n}\to x_{\infty }$ in $W_{0}^{1,p}\left(0,1\right)$, possibly up to subsequence.*

**Proof.**  Take any p≥ρ and denote by $\Lambda :\mathrm{Homeo}\left(R\right)\times {\left(C\left[0,1\right]\right)}^{*}⟶W_{0}^{1,p}\left(0,1\right)$ an operator mapping pairs $\left(\phi ,f^{*}\right)$ into a unique weak solution to (5), which is given by (8). Define the Nemytskii operator $N_{g}:W_{0}^{1,p}\left(0,1\right)\times L^{\infty }\left(\left[0,1\right],U\right)⟶L^{1}\left(0,1\right)$ associated with *g* by

$$N_{g}\left(x,u\right)\left(t\right)=g\left(t,x\left(t\right),x^{\prime }\left(t\right),u\left(t\right)\right)\;\mathrm{for}\;a.e.\;t\in \left[0,1\right].$$

The continuity and boundedness of $N_{g}$ follows form the assumption (10) and Theorem 1. Using the continuity of inclusion $j:L^{1}\left(0,1\right)↪{\left(C\left[0,1\right]\right)}^{*}$ we obtain that the mapping

$$W_{0}^{1,p}\left(0,1\right)\times L^{\infty }\left(\left[0,1\right],U\right)∋\left(x,u\right)⟼j\left(N_{g}\left(x,u\right)\right)\in {\left(C\left[0,1\right]\right)}^{*}$$

is also continuous and bounded on bounded sets. Define a mapping $\Phi :W_{0}^{1,p}\left(0,1\right)\times N_{\infty }⟶W_{0}^{1,p}\left(0,1\right)$ given by

$$\Phi \left(x,n\right)=\Lambda \left(\phi_{n},j\left(N_{g}\left(x,u_{n}\right)\right)-f_{n}^{*}\right)\;\mathrm{for}\;\mathrm{all}\;x\in W_{0}^{1,p}\left(0,1\right)\;\mathrm{and}\;\mathrm{every}\;n\in N_{\infty }.$$

Since sequences $\left(\phi_{n}\right)$, $\left(f_{n}^{*}\right)$, and $\left(u_{n}\right)$ are convergent, and since mappings Λ and $N_{g}$ are continuous, Φ is also continuous. Moreover for every bounded set $B\subset W_{0}^{1,p}\left(0,1\right)$, the boundedness of sequences $\left(f_{n}^{*}\right)$ and $\left(u_{n}\right)$ provides that set $S={⋃}_{n\in N_{\infty }}\left(j\left(N_{g}\left(B,u_{n}\right)\right)-f_{n}^{*}\right)$ is bounded in ${\left(C\left[0,1\right]\right)}^{*}$ and, hence, a relatively weak compact. Theorem 3 provides the continuity of Λ on *S* and, hence, the relative compactness of $\Phi (B\times N_{\infty })$. Therefore, we can use Lemma 1 with $\Sigma =N_{\infty }$, $X=W_{0}^{1,p}\left(0,1\right)$ and A=Φ to get the assertion. □

### Existence and Dependence on Parameters

For $u\in L^{\infty }\left(0,1\right)$ we denote the *essential range* of *u* by

$$ess.ran\left(u\right)=\left\{\xi \in R:\mathrm{for}\;\mathrm{all}\;\varepsilon >0\;a\;\mathrm{set}\;\{t:|u(t)-\xi |<\varepsilon \}\;\mathrm{has}\;a\;\mathrm{positive}\;\mathrm{measure}\right\}$$

and consider the following assumption. The advantage of using the essential range follows from the Example 3.

(12) $$\left.\begin{array}{c} p_{n}\to p_{\infty }\;\mathrm{in}\;\left(1,\infty \right),\\ u_{n}\to u_{\infty }\;\mathrm{in}\;L^{\infty }\left(0,1;U\right),\\ f_{n}^{*}{⇀}^{*}f_{\infty }^{*}\;\mathrm{in}\;{\left(C\left[0,1\right]\right)}^{*}. \end{array}\right\}$$

(13) $$\left.\begin{array}{c} g_{1}:\left[0,1\right]\times R\times U⟶R\;\mathrm{is}\;a\;\mathrm{Carath}é\mathrm{odory}\;\mathrm{function}.\\ \mathrm{For}\;\mathrm{all}\;n\in N_{\infty }\;\mathrm{there}\;\mathrm{exist}\;\alpha_{n}<\lambda_{p_{n}}\;\mathrm{and}\;\beta_{n}\in L^{1}\left(0,1\right)\;\mathrm{such}\;\mathrm{that}\\ \;|g_{1}\left(t,x,v\right)|\leq \alpha_{n}{\left|x\right|}^{p_{n}-1}+\beta_{n}\left(t\right)\\ \mathrm{for}\;a.e.\;t\in \left[0,1\right]\;\mathrm{and}\;\mathrm{all}\;x,v\in R\times ess.ran\left(u_{n}\right). \end{array}\right\}$$

**Theorem** **5.** *Let assumptions *(12)* and *(13)* hold and fix any p such that $p_{n}\geq p$ for all $n\in N_{\infty }$. Then problem*

(14) $$\left\{\begin{array}{c} -{\left(|x^{\prime }{|}^{p_{n}-2}x^{\prime }\right)}^{\prime }=g_{1}\left(t,x\left(t\right),u_{n}\left(t\right)\right)+a_{n}x^{\prime }\left(t\right)+f_{n}^{*},\\ x(0)=x(1)=0, \end{array}\right.$$

*is solvable for every $n\in N_{\infty }$. If additionally $\inf_{n\in N_{\infty }}\left|\lambda_{p_{n}}-\alpha_{n}\right|>0$ and $\left(\beta_{n}\right)$ is bounded in $L^{1}\left(0,1\right)$, then:*

*1*. *if $x_{n}$, n∈N, is a solution to *(14)* we get that $x_{n}\to x$ in $W_{0}^{1,p}\left(0,1\right)$, where x is a solution to *(14)* with n=∞;*
*2*. *if $x⟼g_{1}\left(t,x,u\right)$ is non-increasing for a.e. t∈[0,1] and all u∈U, then *(14)* has a unique solution $\bar{x_{n}}$ for every $n\in N_{\infty }$ and $\bar{x_{n}}\to \bar{x_{\infty }}$ in $W_{0}^{1,p}\left(0,1\right)$.*

**Proof.**  For every $n\in N_{\infty }$ we define the operators $T_{1,n},T_{2,n}:W_{0}^{1,p_{n}}\left(0,1\right)⟶W^{-1,q_{n}}\left(0,1\right)$ by

$$\begin{array}{cc} \langle T_{1,n}\left(x\right),y\rangle & =\int_{0}^{1}{\left|x^{\prime }\left(t\right)\right|}^{p_{n}-2}x^{\prime }\left(t\right)y^{\prime }\left(t\right)dt\\ \langle T_{2,n}\left(x\right),y\rangle & =a_{n}\int_{0}^{1}x^{\prime }\left(t\right)y\left(t\right)dt,\\ \langle T_{3,n}\left(x\right),y\rangle & =\int_{0}^{1}g_{1}\left(t,x\left(t\right),u_{n}\left(t\right)\right)y\left(t\right)dt-\langle f_{n}^{*},y\rangle . \end{array}$$

Let $T_{n}=T_{1,n}-T_{2,n}-T_{3,n}$. Note that *x* is a solution to (14) iff $T_{n}\left(x\right)=0$. Note that using an integrations by parts we obtain

(15) $$\langle T_{2,n}x,x\rangle =a_{n}\int_{0}^{1}x^{\prime }\left(t\right)x\left(t\right)dt=-a_{n}\int_{0}^{1}x\left(t\right)x^{\prime }\left(t\right)dt=0\;\mathrm{for}\;\mathrm{all}\;x\in W_{0}^{1,p}\left(0,1\right).$$

Using both, the Sobolev and the Poincaré inequalities together with assumption (13) we get

$$\begin{array}{cc} \langle T_{n}\left(x\right),x\rangle & =\int_{0}^{1}{\left|x^{\prime }\left(t\right)\right|}^{p_{n}}dt-a_{n}\int_{0}^{1}x^{\prime }\left(t\right)x\left(t\right)dt-\int_{0}^{1}g_{1}\left(t,x\left(t\right),u_{n}\left(t\right)\right)x\left(t\right)dt-\langle f_{n}^{*},x\rangle \\ \; & \geq {∥x∥}_{W_{0}^{1,p_{n}}}^{p_{n}}-\alpha_{n}{∥x∥}_{L_{p_{n}}}^{p_{n}}-∥\beta_{n}{∥}_{L^{1}}{∥x∥}_{W_{0}^{1,p_{n}}}-∥f_{n}^{*}{∥}_{C^{*}}{∥x∥}_{W_{0}^{1,p_{n}}}\\ \; & \geq \left(\frac{\lambda_{p_{n}}-\alpha_{n}}{\lambda_{p_{n}}}{∥x∥}_{W_{0}^{1,p_{n}}}^{p_{n}-1}-∥\beta_{n}{∥}_{L^{1}}-{∥f_{n}^{*}∥}_{C^{*}}\right){∥x∥}_{W_{0}^{1,p}} \end{array}$$

for all $x\in W_{0}^{1,p}\left(0,1\right)$, which implies coercivity of $T_{n}$. Moreover, we see that $W_{0}^{1,p_{n}}$-norm of any solution to (14) is bounded by term $M_{n}:={\left(\frac{\lambda_{p_{n}}}{\lambda_{p_{n}}-\alpha_{n}}\left(∥\beta_{n}{∥}_{L^{1}}+{∥f_{n}^{*}∥}_{C^{*}}\right)\right)}^{q_{n}/p_{n}}$. The monotonicity of $T_{1,n}$ and $T_{2,n}$ can be checked following (15) and [22], while the strongly continuity of $T_{3,n}$ is a consequence of Theorem 1 and compact inclusions $W_{0}^{1,p_{n}}\left(0,1\right)\subset C\left[0,1\right]$, $n\in N_{\infty }$. Therefore, we can use the Browder–Minty Theorem to get the existence of a solution $x_{n}$ to (14) for every $n\in N_{\infty }$. Notice that an additional assumption about the convergence of $\left(\alpha_{n}\right)$ and $\left(\beta_{n}\right)$ provides that $M:=\max_{n\in N_{\infty }}M_{n}<\infty$. Hence we get $∥x_{n}{∥}_{W_{0}^{1,p}}\leq {∥x_{n}∥}_{W_{0}^{1,p_{n}}}\leq M$ and, therefore, we can use Theorem 4 to obtain required convergence $x_{n}\to x$. Finally, notice that the monotonicity of $x⟼g_{1}\left(t,x,u\right)$ implies that

$$\langle T_{n}\left(x\right)-T_{n}\left(y\right),x-y\rangle >0\;\mathrm{for}\;\mathrm{all}\;\mathrm{distinct}\;x,y\in W_{0}^{1,p_{n}}\left(0,1\right)$$

This clearly implies the uniqueness of a solution and finishes the proof. □

**Example** **3.** *Let $p_{n}\to p_{\infty }$ and $\rho_{n}\to \rho_{\infty }$ in [1,∞), $\mu_{n}\to \mu_{\infty }$, and $a_{n}\to a_{\infty }$ in R and $f_{n}⇀f_{\infty }$ in $L^{1}\left(0,1\right)$. Assume that*

(16) $$\left\{\begin{array}{c} -{\left(|x^{\prime }{|}^{p_{n}-2}x^{\prime }\right)}^{\prime }=\mu_{n}{\left|x\right|}^{\rho_{n}-2}x+a_{n}x^{\prime }+f_{n},\\ x(0)=x(1)=0 \end{array}\right.$$

*has a solution for every n∈N. Taking U=[1,∞) and $g\left(t,x,y,u\right)={\left|x\right|}^{u-2}x$ we can apply Theorem 4 to obtain $∥x_{n}{∥}_{W_{0}^{1,1}}\to \infty$ whenever $p_{n}\to p_{\infty }=2$, $\rho_{n}=2$, $a_{n}\to 0$, and $f_{n}⇀f_{0}$ in $L^{1}\left(0,1\right)$, where*

$$f_{0}\left(t\right)=\sin\left(\pi t\right)\;\mathrm{for}\;\mathrm{every}\;t\in \left[0,1\right].$$

*In particular we get $∥x_{n}{∥}_{W_{0}^{1,1}}\to \infty$ for a sequence $\left(x_{n}\right)$ of solutions to *(1)*. On the other hand, if we assume that $p_{n}↘p_{\infty }$, $\rho_{n}\leq p_{n}$, and $\mu_{n}<\lambda_{p_{n}}$ for all $n\in N_{\infty }$, then problem *(16)* has a solution $x_{n}$ for all n∈N. Moreover, $x_{n}\to x_{\infty }$ in $W_{0}^{1,p_{\infty }}\left(0,1\right)$ (up to subsequence), where $x_{\infty }$ is a solution to *(16)* (with n=∞).*

Allowing for $\lambda_{n}↘\lambda_{\infty }<\pi^{2}$ we do not meet the problems encountered in Example 1.

## 5. Discussion

This research provides additional advanced and complex information concerning the Hadamard Programme about non-linear equations. Our input relies on the fact that apart to standard parameter dependence we also allow for some structure stability with respect to the differential operator which is allowed to vary as far as it is still an increasing homeomorphism. The state of the art pieces concerned either the sole dependence on functional parameters (also incorporating some information whether the solution is of variational type which is not that important if one knows that this is a solution) or else these were concerned on the asymptotic analysis of the operator with reference to its eigenvalues. Our approach was to somehow coin the two approaches and allow the functional parameter to vary together with the differential operator. The second advance with reference to the dependence on parameters is that now we allow for quite a general type of parameters, i.e., the Dirac delta where the information can somehow be packed in one particle and, thus, leading a way to applications in physics. In the sources mentioned in the bibliography and also in those which cite them, the parameters are assumed rather regular. The methods pertain to the fixed point and monotone ones indicating that a possible further impact is also possible if one tries, with a modified approach, to use operators with more general monotonicity than the increasing homeomorphism.

## Data Availability

Not applicable.

## Abbreviations

The following abbreviations are used in this manuscript:

MDPI	Multidisciplinary Digital Publishing Institute
DOAJ	Directory of open access journals
TLA	Three letter acronym
LD	Linear dichroism
